# Role of PI3 Kinases in Cell Signaling and Soleus Muscle Atrophy During Three Days of Unloading

**DOI:** 10.3390/ijms26010414

**Published:** 2025-01-06

**Authors:** Ksenia A. Zaripova, Svetlana P. Belova, Tatiana Y. Kostrominova, Boris S. Shenkman, Tatiana L. Nemirovskaya

**Affiliations:** 1 Myology Laboratory, Institute of Biomedical Problems (IBP), RAS, 123007 Moscow, Russia; katsu.no.himitsu@gmail.com (K.A.Z.); swetbell@mail.ru (S.P.B.);; 2 Department of Anatomy, Cell Biology and Physiology, Indiana University School of Medicine-Northwest, Gary, IN 46202, USA; tkostrom@iu.edu

**Keywords:** PI3 kinase, muscle atrophy, unloading, ATP, MuRF1

## Abstract

During skeletal muscle unloading, phosphoinositide 3-kinase (PI3K), and especially PI3K gamma (PI3Kγ), can be activated by changes in membrane potential. Activated IP3 can increase the ability of Ca^2+^ to enter the nucleus through IP3 receptors. This may contribute to the activation of transcription factors that initiate muscle atrophy processes. LY294002 inhibitor was used to study the role of PI3K in the ATP-dependent regulation of skeletal muscle signaling during three days of unloading. Inhibition of PI3K during soleus muscle unloading slows down the atrophic processes and prevents the accumulation of ATP and the expression of the E3 ubiquitin ligase MuRF1 and ubiquitin. It also prevents the increase in the expression of IP3 receptors and regulates the activity of Ca^2+^-dependent signaling pathways by reducing the mRNA expression of the Ca^2+^-dependent marker calcineurin (CaN) and decreasing the phosphorylation of CaMKII. It also affects the regulation of markers of anabolic signaling in unloaded muscles: IRS1 and 4E-BP. PI3K is an important mediator of skeletal muscle atrophy during unloading. Developing strategies for the localized skeletal muscle release of PI3K inhibitors might be one of the future treatments for inactivity and disease-induced muscle atrophy.

## 1. Introduction

Changes in intracellular signaling pathways occur within hours/days of muscle unloading [1]. It is known that during skeletal muscle unloading, there is an increased accumulation of high-energy phosphates (ATP, PCr) and Ca^2+^ ions [2,3,4]. One of the earliest effects of unloading on the postural muscle is depolarization of the sarcolemma due to inactivation of the α2 subunit of Na,K-ATPase [5,6]. Membrane depolarization develops within several hours of unloading and precedes the apparent signs of skeletal muscle atrophy [6]. Decreased resting membrane potential is accompanied by the accumulation of Ca^2+^ in the sarcoplasm [2,4]. Dihydropteridine receptor (DHPR) and PANX1 channels regulate the release of ATP into extracellular space [7].

It was previously reported that ATP can trigger gene activation and/or repression during unloading [3,8]. It was suggested that unloading initiates changes in plasma membrane potential and leads to the activation of Ca^2+^-dependent DHPR channels located near the PANX1 channels. Animal muscle cell culture experiments showed that increased accumulation of ATP in the extracellular space can delay the slowdown of Ca^2+^ release in myoplasm via the P2Y2-PLC-IP3 pathway [7,9]. We suggest that similar mechanisms of activation of the IP3-dependent pathway may be involved in the mediation of unloading-induced signaling. This could be related to the previously described processes where inhibition of DHPR and pharmacologically induced decrease in Ca^2+^ and high-energy phosphates in unloaded soleus muscle diminished negative metabolic changes [4,10]. The complete mechanisms involved in these processes have not been fully elucidated.

**Figure 1:**
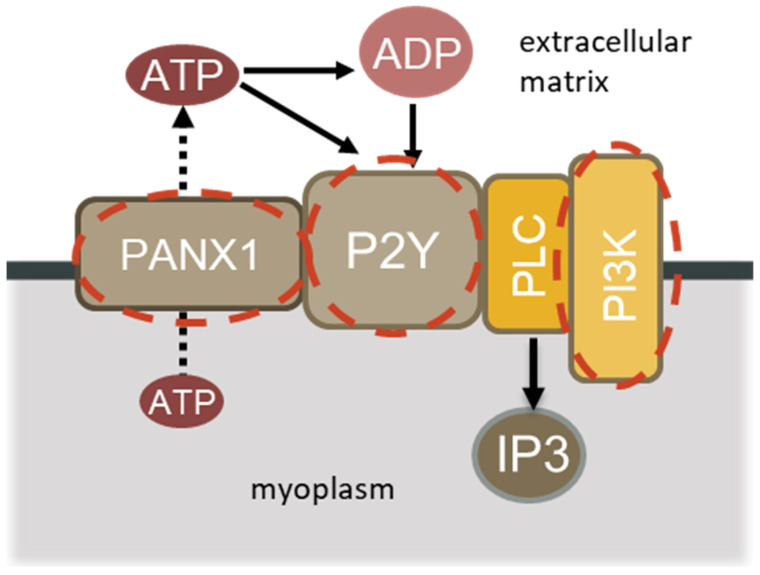
Schematic summarizing the signaling resulting in PI3 kinase activation.

It was previously reported that membrane depolarization activates pannexin channels (PANX1), which allow ATP to exit muscle fibers [7,11]. Inhibition of PANX1 during unloading changes the rate of ATP transport from muscle fibers and prevents many negative effects of unloading [3]. Opening of PANX1 channels might have several potential effects on muscle atrophy. ATP leaves muscle fibers through pannexin channels and interacts with P2Y1/2 purinergic receptors [12]. Purinergic receptors modify signaling and activate atrophic processes during muscle unloading [13]. P2Y1/2 receptors activate PI3 kinase gamma (PI3Kγ) and, ultimately, IP3R, located in the nucleus and the sarcoplasmic reticulum (Figure 1).

It is known that activating purinergic receptors by exogenous agonists causes Ca^2+^ release, which depends on the IP3 signaling pathway [14]. Purinergic agonists have previously been shown to activate PI3Kγ preferentially [14,15]. Both Wortmannin and LY294002 prevent tyrosine phosphorylation and membrane translocation of PLCγ, as well as IP3 generation in ATP-stimulated cells [15]. IP3 is produced by the binding of ATP to purinergic receptors located on the outer side of the sarcolemma [15,16]. Activation of IP3 receptors (IP3Rs) can induce a weak signal for calcium release in sarcoplasm and nucleoplasm, and this activates transcription factors resulting in the expression or repression of muscle genes [17].

The release of Ca^2+^ caused by the depolarization of skeletal muscle cells can be divided into two independent processes: “fast” transient Ca^2+^, uniformly distributed throughout the cell, corresponding to excitation–contraction coupling, and “slow” transient Ca^2+^ with a distinct nuclear component generated by IP3 [18,19]. “Slow” Ca^2+^ can stimulate the activation of intracellular signaling pathways and is involved in the coupling of excitation and transcription [20,21,22]. The term “slow” Ca^2+^ refers to the increased concentration of Ca^2+^ that is not associated with contraction and remains in the sarcoplasm for a long time. It peaks after 60–100 s and is mostly associated with cell nuclei. It was reported, using cultured rodent myotubes, that slow calcium processes are mediated by IP3 but not by RYR [23,24].

Phosphatidylinositol 3-kinases (PI3Ks) have previously been reported to be involved in regulating the balance between protein synthesis and protein degradation during disuse-induced muscle atrophy [25]. In addition to their catalytic activity, class I PI3K catalytic subunits have protein scaffolding functions that depend on protein–protein interactions and not on their enzymatic activity [26,27,28,29,30]. A key function of the scaffold is the stabilization of proteins bound to PI3K, and this function is independent of the catalytic activity of PI3K. This explains why approaches to knockout or knockdown of the PI3K gene often result in the attenuated expression of not only the PI3Ks but also their binding partners. This scaffolding role of PI3K hinders the predictive potential of reducing its expression levels to model pharmacological effects by these protein kinases. On the contrary, PI3K inhibitors inactivate PI3K catalytic activity without affecting PI3K protein expression and their scaffolding properties as enhancers of protein–protein interactions [26,27,28,29,30,31]. Therefore, it is important to note the two different roles of PI3K: catalytic and scaffolding [31].

We suggested that activation of DHPR (L-type calcium channels) and depolarization of the sarcolemma during unloading may act as a voltage sensor activating the phospholipase C/inositol 1,4,5-triphosphate-dependent signaling pathway (Figure 1). Our suggestion was based on previously published studies [19,32]. It was reported, using cultured rat myotubes, that G protein and PI3K were activated by electrical stimulation [32], and the increase in inositol 1,4,5-trisphosphate and the “slow” Ca^2+^ signal induced by electrical stimulation were blocked by PI3K inhibitors [19]. The authors showed that the Gβγ/PI3Kγ signaling pathway is involved in the activation of phospholipase C and the generation of a “slow” Ca^2+^ signal. Furthermore, Yang et al. reported an increase in the content of IP3, as well as IP3R1 and Ca^2+^ (cytoplasmic and nuclear) in the unloaded soleus muscle of rats [33]. The authors suggest that increased levels of IP3 and Ca^2+^ during unloading contribute to the activation of IP3R1 and an increase in the concentration of Ca^2+^ in the nucleus.

We hypothesized that PI3K (in particular PI3Kγ) may be activated by changes in the membrane potential during muscle unloading, as a result of which IP3 may increase the ability of Ca^2+^ to enter the nucleus through IP3Rs. Along with some other signaling pathways [1,34], the activation of IP3Rs may contribute to the activation of transcription factors and stimulate the initiation of atrophic processes during muscle unloading. Currently, there are no publications on the role of PI3K in the regulation of signaling processes during muscle unloading. However, the role of PI3K inhibition in protecting the heart muscle was previously reported [35]. The rationale of the current study was to evaluate whether the changes in IP3 receptor function caused by PI3K inhibition during muscle unloading would prevent or significantly reduce the expression of E3 ligases and interfere with the activation of transcription factors that regulate muscle atrophy. To test the hypothesis, we performed a three-day muscle unloading experiment evaluating the soleus muscle of rats with and without treatment with PI3K inhibitor LY294002. The aim was to evaluate the early stages of muscle unloading to elucidate the mechanisms and to develop an effective prophylactic intervention for muscle atrophy. We chose three days of unloading since substantial changes in signaling can already be observed at this time, but there are no significant slow-to-fast changes in the contractile proteins [34]. The changes in the slow-to-fast shift and abnormalities in the contractile proteins are readily observed at seven days of unloading [36].

## 2. Results

### 2.1. The Effect of PI3K Inhibition with LY294002 on Unloaded Muscle Mass

**Figure 2:**
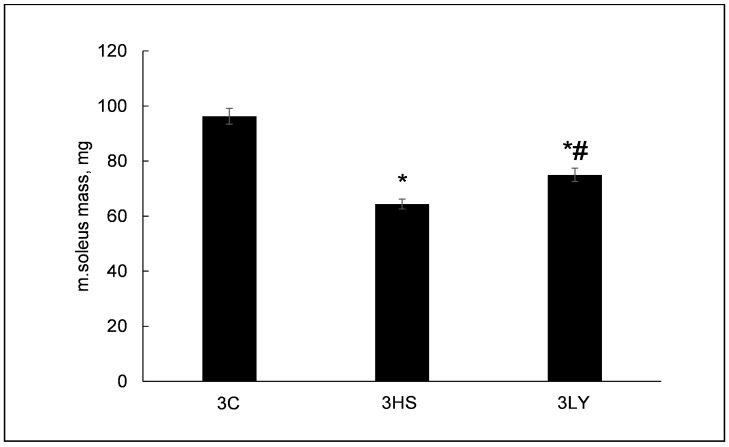
Evaluation of soleus muscle mass in control (3C), three-day unloaded (3HS), and LY294002-treated three-day unloaded (3LY) rats. N = 8. * indicates a significant difference from the control, and # indicates a significant difference from the 3HS group, *p* < 0.05.

Three days of unloading significantly decreased soleus muscle mass in the 3HS and 3LY groups (64.4 ± 1.7 mg *p* < 0.0001 and 74.9 ± 2.4 mg *p* < 0.0002, respectively) compared with the control (96.3 ± 2.8 mg) (Figure 2). Treatment with LY294002 diminished the unloading-induced muscle decline, reaching a statistical significance between the 3HS and 3LY groups (*p* < 0.004; Figure 2).

### 2.2. The Effect of PI3K Inhibition with LY294002 on Phospho-PI3K, IP3, ATP, and Phospho-AMPK Content in Unloaded Muscle

**Figure 3:**
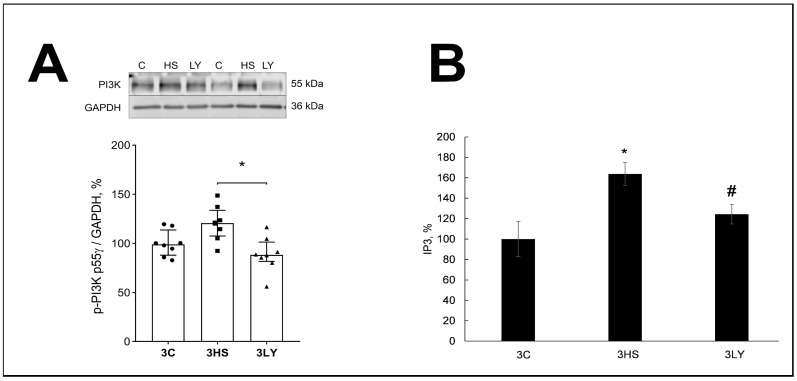
Evaluation of phospho-PI3Kγ (**A**) and IP3 (**B**) contents in the muscles of control (3C), three-day unloaded (3HS), and LY294002-treated three-day unloaded (3LY) rats. The level of phosphorylated PI3K (p-PI3K) was normalized to the level of GAPDH in each sample (**A**). N = 8. * indicates a significant difference from the control, and # indicates a significant difference from the 3HS group, *p* < 0.05.

The content of phospho-PI3K was elevated and IP3 was significantly higher in the 3HS group compared with the 3C and 3LY groups (*p* < 0.03 and *p* < 0.003; Figure 3). This suggests that LY294002 was effective in inhibiting PI3K activation during soleus muscle unloading.

**Figure 4:**
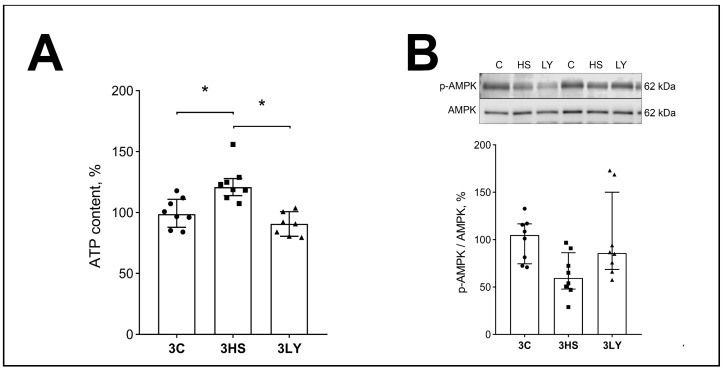
Evaluation of ATP (**A**) and p-AMPK (**B**) contents in the muscles of control (3C), three-day unloaded (3HS), and LY294002-treated three-day unloaded (3LY) rats. Phosphorylated AMPK (p-AMPK) was normalized to the total AMPK content in each sample (**B**). N = 8. * indicates a significant difference, *p* < 0.05.

Compared with the control, the ATP content was increased in the 3HS group (*p* < 0.007; Figure 4A). Inhibition of PI3K during soleus muscle unloading prevented the accumulation of ATP (*p* < 0.001; 3LY group), and it did not differ from the 3C group (Figure 4A). On the contrary, there was a trend for the decrease in the content of phospho-AMPK in unloaded muscle (3HS group; Figure 4B). Inhibition of PI3K during soleus muscle unloading prevented this decrease (3LY group; Figure 4B).

### 2.3. The Effect of PI3K Inhibition with LY294002 on Ca^2+^ Signaling and IP3R Content in Unloaded Muscle

**Figure 5:**
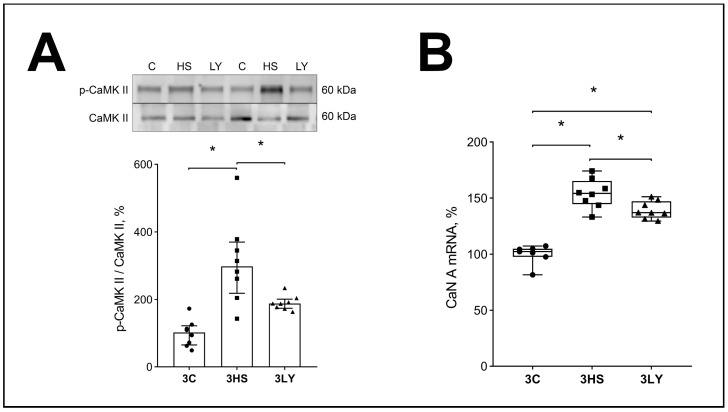
Evaluation of phospho-CaMKII content (**A**) and CaN mRNA expression (**B**) in the muscles of control (3C), three-day unloaded (3HS), and LY294002-treated three-day unloaded (3LY) rats. Phosphorylated CaMKII was normalized to the total CaMKII content in each sample (**A**). N = 8. * indicates a significant difference, *p* < 0.05.

The content of phosphorylated CaMKIIb was increased in the unloaded soleus muscle (3HS group) relative to the control group (*p* < 0.005; Figure 5A). Administration of the PI3K inhibitor LY294002 prevented these changes (*p* < 0.005, 3LY group versus 3HS group; Figure 5A).

Similar results were obtained for the CaN mRNA content (*p* < 0.0001 and *p* < 0.02 for the 3HS group and 3LY group versus the 3HS group; Figure 5B).

**Figure 6:**
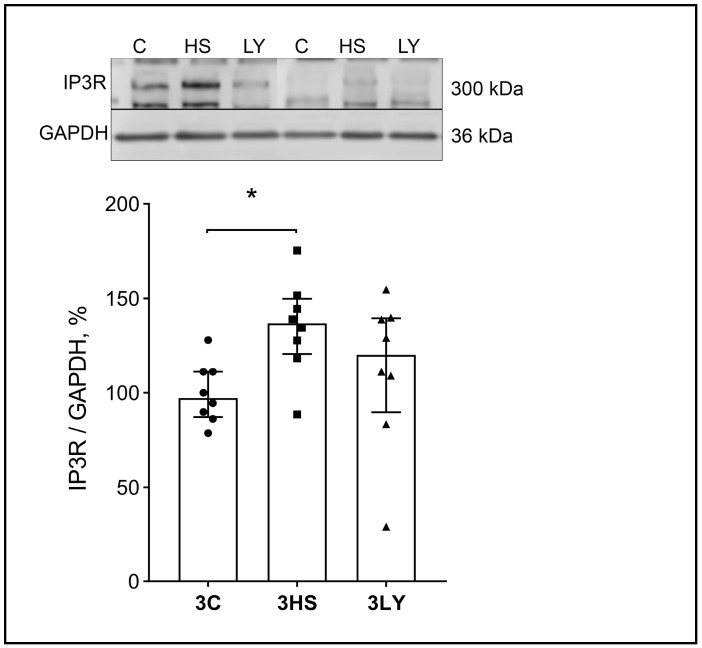
Evaluation of IP3R content in the muscles of control (3C), three-day unloaded (3HS), and LY294002-treated three-day unloaded (3LY) rats. The content of IP3R was normalized to the GAPDH content in each sample. N = 8. * indicates a significant difference, *p* < 0.005.

The content of IP3R in the 3LY group was also significantly lower than in the unloaded group without LY294002 administration (*p* < 0.005; Figure 6).

### 2.4. The Effect of PI3K Inhibition with LY294002 on Markers of Catabolic Signaling in Unloaded Muscle

**Figure 7:**
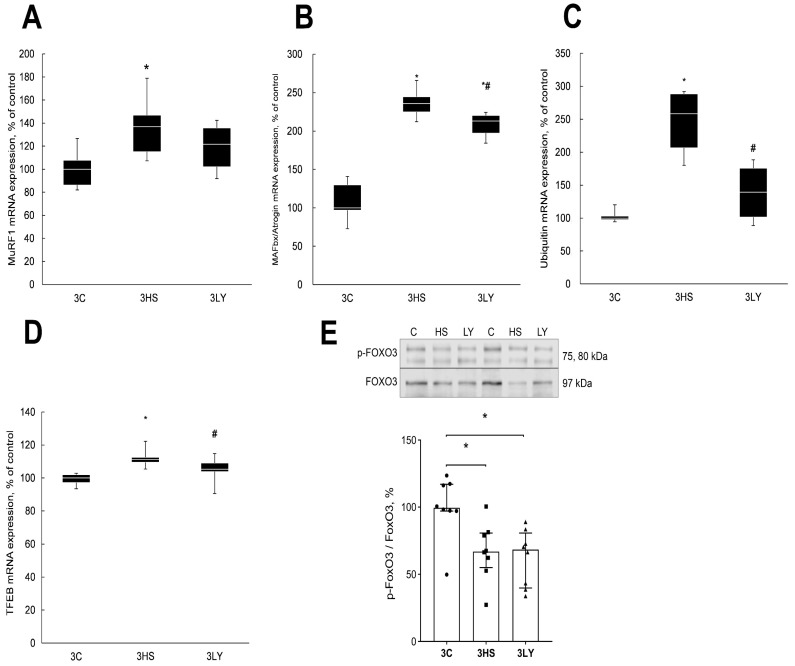
Evaluation of MuRF1 (**A**), MAFbx (**B**), ubiquitin (**C**), and TFEB (**D**) mRNA expression and phospho-FOXO3 (**E**) content in the muscles of control (3C), three-day unloaded (3HS), and LY294002-treated three-day unloaded (3LY) rats. Phosphorylated FOXO3 content was normalized to the total FOXO3 content in each sample (**E**). N = 8. * indicates a significant difference from the control, and # indicates a significant difference from the 3HS group, *p* < 0.05.

In the 3HS group, the level of mRNA expression of E3 ligases MuRF1, MAFbx, and ubiquitin was significantly higher than in the control group (*p* < 0.003 and *p* < 0.0001; Figure 7). Treatment with the PI3K inhibitor LY294002 prevented the increase in the expression of MuRF1 and ubiquitin mRNA in the 3LY group (Figure 7A,C) and significantly reduced the expression of MAFbx (*p* < 0.005; 3LY; Figure 7B).

Transcription factor EB (TFEB) is a known regulator of MAFbx and MuRF1 expression during unloading [37]. TFEB content was increased in the 3HS group, and treatment with LY294002 prevented the increase (*p* < 0.0001; Figure 7D).

The content of phosphorylated FoxO3 was decreased in the 3HS group compared with the control (*p* < 0.01; Figure 7E). Treatment with PI3K inhibitor had no statistically significant effect on the content of phosphorylated FoxO3 during unloading (3LY group; Figure 7E).

### 2.5. The Effect of PI3K Inhibition with LY294002 on Markers of Anabolic Signaling in Unloaded Muscle

**Figure 8:**
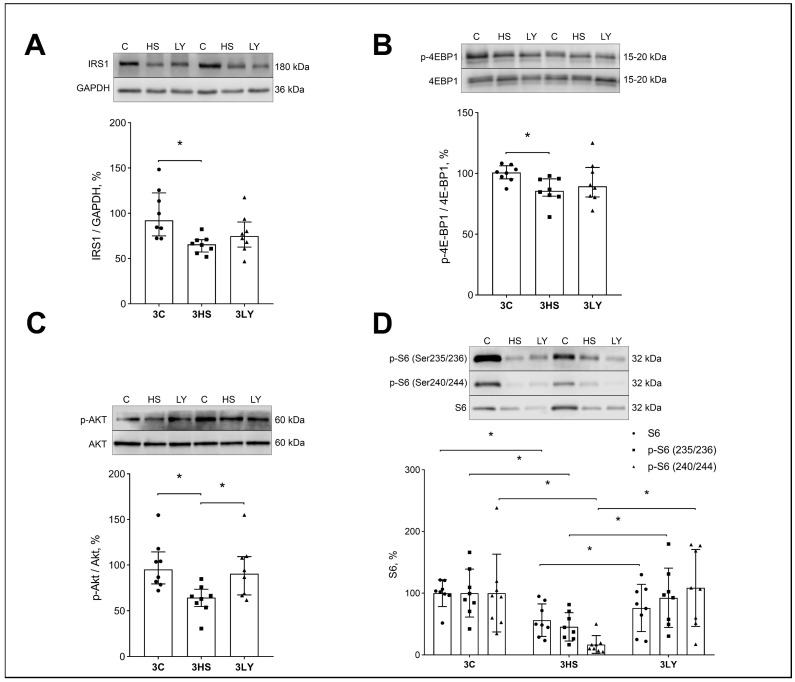
Evaluation of IRS1 (**A**), phospho-4EBP1 (**B**), phospho-Akt (**C**), and total/phospho-S6 (**D**) contents in the muscles of control (3C), three-day unloaded (3HS), and LY294002-treated three-day unloaded (3LY) rats. IRS1 content was normalized to the content of GAPDH in each sample (**A**). Phosphorylated 4EBP1, Akt, and S6 contents were normalized to the total 4EBP1, Akt, and S6 contents in each sample (**B**). N = 8. * indicates a significant difference, *p* < 0.05.

Inhibition of PI3K during unloading prevents the decrease in the expression of markers of protein synthesis signaling pathways. Unloading significantly reduced the expression and/or phosphorylation of protein synthesis markers IRS-1 (*p* < 0.008; Figure 8A), phospho-4E-BP (*p* < 0.008; Figure 8B), phospho-Akt (Figure 8C), and phospho-S6 (*p* < 0.005; Figure 8D) in the 3HS group. Treatment with the LY294002 inhibitor prevented these changes, and the values in the 3LY group were not different from those in the 3C group.

## 3. Discussion

The aim of the current study was to evaluate whether the changes in IP3 receptor function caused by PI3K inhibition would prevent the expression of E3 ligases and interfere with the activation of transcription factors that regulate unloading-induced muscle atrophy. In the current study, the three days of unloading of the soleus muscle results in atrophy; this correlates well with previously reported data [3,13]. Treatment with PI3K inhibitor LY294002 decreased the degree of unloading-induced muscle atrophy compared to the unloaded group without treatment. A decrease in the rate of atrophy can occur both by slowing down the rate of catabolic processes as well as by preventing a decline in the rate of anabolic processes [38].

We found that the level of PI3K phosphorylation and the level of IP3 in the 3LY group was similar to the control group and was significantly reduced compared to the 3HS group. An increase in rat soleus IP3 content during unloading was previously reported [33]. The result may indicate an adequate effect of the LY294002 inhibitor on the functioning of PI3K. It was previously noted that PI3K activation depends on the balance of activated isoforms (PI3Kα and PI3Kγ), and it can have a different effect (beneficial or detrimental) depending on the specific PI3K isoform that is activated [39].

### 3.1. Effect of LY294002 Administration on the Content of ATP and AMPK in the Soleus Muscle of Rats

Early during unloading (1–3 days), there is an increase in the level of high-energy phosphates in muscle [3]. This period is characterized by an essentially complete absence of electrical and mechanical activity recorded in the muscle [40]. It is reasonable that under these conditions, in the absence of contractile activity and the absence of significant impairments in the activity of mitochondria, there is an increase in muscle ATP content. PI3Kγ is located in the sarcolemma and is integrated into signaling cascades associated with the activation of P2Y1/2 receptors. It was previously reported that during muscle unloading, pannexin channels can participate in the efflux of ATP from muscle fibers [3], and P2Y1/2 receptors can participate in the regulation of this signaling [13]. It can be suggested that inhibition of PI3K activity can affect signaling from ATP-activated purinergic receptors, and this can affect the ATP content in the muscle.

The level of phospho-AMPK in the 3HS group was not significantly reduced relative to the control group in the current study. Interestingly, a different study showed a significant decrease in this parameter in the soleus muscle during 3 days of unloading [13]. It is known that ATP is specifically regulated by Ca^2+^ ions in slow muscles like soleus [41].

### 3.2. Effect of LY294002 Administration on Ca^2+^ Signaling

IP3 is an important second messenger that regulates Ca^2+^ homeostasis in the cell [42]. We measured the levels of markers of Ca^2+^-dependent signaling: CaMK IIb and CaN. CaMKII is a calcium-dependent kinase [43]. During unloading, intracellular Ca^2+^ concentration increases in both the myoplasm and the cell nucleus [2,33]. CaMKII activity is regulated by intracellular Ca^2+^ [44,45]. In the present study, the phosphorylation of CaMKIIb was increased in the unloaded soleus muscle (3HS group) relative to the control group, but the administration of the PI3K inhibitor LY294002 prevented these changes. Phosphorylation of several proteins is regulated by CaMKII, including AMPK [45,46] and several transcription factors [47].

For CaN, observations were similar to the results on CaMK IIb. CaN expression in the soleus muscle of the 3LY group was significantly lower than in the unloaded group without the LY294002 inhibitor. CaN is a Ca^2+^- and calmodulin-dependent phosphatase, the activity of which increases with increasing Ca^2+^ concentration in muscles [48,49,50]. The actions of CaN and CaMKII are interconnected [51]. Dephosphorylation of proteins by CaN can initiate their translocation into the nucleus. There is also an obvious association between the inhibition of PI3K, the level of its phosphorylation, and the content of IP3R in the soleus muscle. IP3Rs are Ca^2+^-dependent receptors that are activated when the concentration of Ca^2+^ in the cytoplasm increases. IP3R passes Ca^2+^ into the nuclei [33]. IP3R level is elevated in the unloaded soleus muscle [33]. Similar observations were made in the current study, and the PI3K inhibitor prevented these changes. The results indicate that PI3K inhibition with LY294002 affects CaN expression and phospho-CaMK IIb and PI3K levels. This observation suggests that PI3K can take part in the regulation of Ca^2+^-dependent signaling pathways during unloading. These changes may be associated with the impediment of increased levels of cytoplasmic Ca^2+^ in the unloaded muscles of rats treated with LY294002.

### 3.3. Effect of LY294002 Administration on the Level of Muscle Catabolic Signaling Markers During Unloading

In the nucleus, Ca^2+^ activates transcription factors that trigger the expression of atrogenes [7,41]. In the present study, the level of mRNA expression of E3 ligase MuRF1, MAFbx, and ubiquitin in the 3HS group was significantly higher than in the control group. Treatment with the PI3K inhibitor prevented the upregulation of these catabolic markers.

The level of FOXO3 transcription factor phosphorylation was comparably reduced in both the 3HS and 3LY groups. This suggests that the effect of the LY294002 inhibitor on the expression of E3 ligases in unloaded muscle was not regulated by FOXO3. Concurrently, the level of Akt phosphorylation in the 3HS group was significantly reduced, while its level in the 3LY group did not differ from the control group. It should be noted that FOXO3 phosphorylation can be regulated not only by Akt but also by several other kinases [52]. Similar results were previously reported for the unloading soleus muscle of rats [53,54].

The expression of another transcription factor TFEB, a known regulator of MAFbx and MuRF1 expression during unloading [37], was significantly different from the control group in the 3HS group but was similar to the control value in the 3LY group. The expression of E3 ligases is regulated by multiple transcription factors [37]. The decrease in the degree of soleus muscle atrophy in the 3LY group could be associated with a TFEB-mediated decrease in protein ubiquitination and protein degradation.

### 3.4. Effect of LY294002 Administration on the Level of Anabolic Signaling Markers

Anabolic processes can be regulated at the level of translation initiation and translation elongation. The current study measured several markers that allow assessment of the direction of these processes. IRS1/AKT/mTORC1 is a canonical signaling pathway that regulates protein synthesis at the level of translation initiation. Akt is a connection between the cell surface receptors coupled to PI3K and the downstream signaling pathways controlling anabolic processes. It is unclear how the Akt activity remains proportional to the upstream signaling. Uncoupling extracellular signals from PI3K activation can result in Akt hyperactivation [55]. However, in our experiments, the level of IRS1 in the 3HS group was reduced, and PI3K was increased relative to the control group. The downregulation of IRS1 during soleus muscle unloading was previously described [56].

There are several isoforms of PI3Ks. The contradiction between the decrease in the level of IRS1 in our experiment and the high content of PI3K in the soleus muscle of 3HS rats may be due to the fact that PLCγ, located in the sarcolemma, and a high level of ATP can activate phosphatidylinositol 3-kinase (PI3Kγ). This mechanism was described previously [32,57]. Activation of PLCγ occurs during membrane depolarization [19], which is also observed during muscle unloading in rats.

Unloading results in decreased Akt phosphorylation [53,58]. We found the same decrease in the present study, which was prevented by the treatment with LY294002. Phosphorylation of Akt can occur not only via IRS-1 but also with the participation of mTORc2 [59,60]. Similar signaling could have been involved in his study. Phospho-Akt can phosphorylate several proteins in the mTORC1 signaling pathway. mTORC1 phosphorylates its two main substrates, S6K1 Ribosomal kinase (Ser235, Ser236, Ser240, and Ser244) and eukaryotic initiation Factor 4E (eIF4E)-binding proteins 1 and 2 (4E-BP1/2) [61]. A decrease in the expression level of markers responsible for translation initiation during 3-day muscle unloading was previously reported [56,62]. In the current study, LY294002 administration activated the Akt-mTOR pathway as evidenced by the significant increase in Akt phosphorylation (Thr308 and Ser473), along with a significant increase in eukaryotic initiation factor 4E-binding protein 1 (4E-BP1) (Thr37/46) and ribosomal protein S6 (Ser235/236) phosphorylation. In the 3HS group, these three markers were significantly reduced compared to the control group.

The elongation factor eEF2 and its kinase (eEF2K) are important signaling proteins that can be regulated independently of mTORC1 [1]. As in previous studies [13], the level of p-eEF2 was increased in the 3HS group relative to the 3C group, which leads to the inhibition of elongation [63]. Inhibition of PI3K prevented the increase in p-eEF2 phosphorylation. The eEF2 phosphorylation is regulated by the quantity and activation status of eEF2K. Excess Ca^2+^/CaN can promote phosphorylation of eEF2 on the Thr56 site by the Ca^2+^/CaN-dependent eEF2K [63], and this correlates with the elongation of the peptide chain on the ribosome; therefore, the increased accumulation of Ca^2+^ in the sarcoplasm can be a trigger for the activation of this signaling pathway [1]. In the current study, the decrease in the level of soleus muscle atrophy in the 3LY group may be associated with the inhibition of a decline in the level of markers of the anabolic pathway at the level of translation initiation as well as translation elongation.

This study has several limitations. The CDN1163 effects were evaluated only in male rats. It is known that unloading can have different effects on the soleus muscle of male and female rats [64]. This is something that we plan to evaluate in future experiments. Another limitation is that only the soleus muscle was evaluated in the current study. The soleus muscle was selected based on the previous observations that three days after unloading, only slow muscles like the soleus were affected, while unloading-induced changes in fast muscles were observed at later time points [64,65].

## 4. Materials and Methods

### 4.1. Animal Protocol Approval

The experiments reported in this study were carried on at the IBMP, RAS, Russia, and complied with the ARRIVE guidelines [66] and rules of biomedical ethics [67]. Animal experiments were reviewed and approved by the Committee on Bioethics, RAS (protocol 617; 22 June 2022).

### 4.2. Animal Procedures

Male Wistar rats (2 months old; 8 animals per group) were assigned at random to one of the three groups: control with placebo (3C; intraperitoneal injection of 400 mL of 10% DMSO, 10% Tween 80 in physiological saline), unloading for 3 days injected with placebo (3HS), unloading for 3 days injected with PI3K inhibitor LY294002 (3LY; 30 mg/kg of body weight per day, intraperitoneally injected with 400 μL of 10% DMSO, 10% Tween 80 in physiological saline). The preliminary experiments were performed using LY294002 concentrations ranging from 10 to 75 mg/kg. The lowest LY294002 concentration (30 mg/kg) that statistically significantly and reliably decreases PI3K phosphorylation was selected for all subsequent experiments.

A tail-casting method was used for hindlimb suspension [68,69]. The rat’s tail was attached with adhesive tape via swivel to a metal bar on the top of a cage. The angle between the rat’s torso and the floor of the cage was about 30°. The hindlimbs of the rats did not touch the floor [38]. Rats were free to move freely around the cage using their forelimbs. During 3 days of unloading, rats had food and water ad libitum. There were no significant differences between the groups in food and water intake or rat cage movement activity. Isoflurane inhalation anesthesia followed by cervical dislocation was used for euthanizing at the end of the animal study. After 3 days of unloading, soleus muscle was frozen in liquid nitrogen and stored at minus 80 degrees Celsius.

### 4.3. Western Blotting

Fifteen milligrams of the mid-belly region of each soleus muscle was homogenized in RIPA buffer (#sc-24948, Santa Cruz Biotechnology, Dallas, TX, USA). The samples were centrifuged at 12,000× *g* for 15 min and the supernatant was collected. Protein concentration was determined using the Bradford reagent and an Epoch plate spectrophotometer (Bio-Tek Instruments, Winooski, VT, USA). Electrophoresis and Western blotting protocols were similar to those previously described [13].

The following primary antibodies were used: IP3 receptors (1:500, #PA5-96855), phospho-CaMKII (Thr286) (1:500, #PA1-4614), phospho-FoxO3 (Ser253) (1:1000, #PA5-37578), and FoxO3 (1:1000, #PA5-20973) from Thermo Fisher Scientific, Waltham, MA, USA. Phospho-PI3K (p-PI3K, 1:500, #CSB-PA030058) from Cusabio, Houston, TX, USA. Antibodies against CaMKII (1:1000, #3362), AMPK (1:1000, ##2532), phospho-AMPK (Thr172) (1:500, #2535), 4E-BP1 (1:1000, #9452), phospho-4E-BP1 (Tr37/46) (1:1000, #2855), Akt (1:1000, #9272), phospho-Akt (Ser473) (1:1000, #9271) IRS-1 (1:1000, #2382), GAPDH (1:5000, #2118), phospho-S6(Ser235/236) (1:1000, #4858), phospho-S6(Ser240/244) (1:1000, #5364), and S6 (1:1000, #2217) were from Cell Signaling Technology, Danvers, MA, USA.

### 4.4. Muscle ATP Content Evaluation

The previously described protocol was used for ATP evaluation and quantification [3,13]. ATP Colorimetric/Fluorometric Assay Kit (MAK190; Sigma, St. Louis, MO, USA) was used to evaluate the ATP content in frozen soleus muscle samples from each rat.

### 4.5. Muscle IP3 Content Evaluation

The IP3 content in muscle lysates was determined using IP3 ELISA Kit #EU3119 from FineTest Biotech Inc. (Wuhan, China), according to the manufacturer’s protocol.

### 4.6. RNA Isolation and Reverse Transcription

RNA isolation and RT were performed using RNeasy Micro Kit (Qiagen, Hilden, Germany) as previously described [13]. RevertAid RT Kit (Thermo Fisher Scientific, USA) was used for RT of 0.5 micrograms of total RNA.

### 4.7. Quantitative PCR Analysis

Amplification was performed using SYBR Green Master Mix (Syntol, Moscow, Russia) and 10 pM of each forward and reverse primer, as previously described [13]. Sequences of the primers synthesized by Syntol (Moscow, Russia) are presented in Table 1. Relative quantification was performed as previously described [25]. RPL19 was used as a reference gene for normalization.

### 4.8. Statistical Evaluation

Statistical analyses were performed using REST 2009 v.2.0.12 (Qiagen, Hilden, Germany) and OpenOffice Calc v. 4.1.13 (OpenOffice.org, www.openoffice.org/product/calc.html (accessed on 31 December 2024)) programs as previously described [13]. The significance of differences between groups was determined using the Kruskal–Wallis test. In the current study, Western blot data are expressed as means ± SD and PCR data as median and interquartile range. *p* < 0.05 was considered a statistically significant difference.

## 5. Conclusions

At the beginning of the study, our hypothesis was that during muscle unloading, PI3K is activated by changes in the membrane potential. As a result, IP3 may increase the ability of Ca^2+^ to enter the nucleus through IP3R. The results of our experiments showed that inhibition of PI3K during three days of muscle unloading prevents ATP accumulation, attenuates muscle atrophy via decreased expression of MuRF1 and ubiquitin, prevents the increase in the expression of IP3 receptors, alters the activity of Ca^2+^-dependent signaling pathways by reducing expression of CaN and by decreased CaMKII phosphorylation, and blocks the reduction in anabolic signaling markers. Based on the results of the study, it can be concluded that IP3K regulates atrophy and atrophic signaling in rat soleus muscle during three days of unloading. The modulation of IP3K function can be regulated by IRS1-dependent signaling and by changes in the markers in the sarcolemma (DHPR/PANX/P2Y1/2R).

## Figures and Tables

**Table 1:** Sequences of primers used in the experiments.

Gene Description	Primer Sequence
**CaN**	5′-GCACACATAGATGGTCGGC-3′ 5′-CAGGTGCATGCTTTGATCGC-3′
**MAFbx**	5′-CTACGATGTTGCAGCCAAGA-3′ 5′-GGCAGTCGAGAAGTCCAGTC-3′
**MuRF-1**	5′-GCCAATTTGGTGCTTTTTGT-3′ 5′-AAATTCAGTCCTCTCCCCGT-3′
**RPL19**	5′-GTACCCTTCCTCTTCCCTATGC-3′ 5′-CAATGCCAACTCTCGTCAACAG-3′
**TFEB**	5′-CCTTCCCCATCATAGGACTGC-3′ 5′-TTTGGACTTAGTGCCTGCCTGG-3′
**Ubiquitin**	5′-CACCAAGAAGGTCAAACAGGA-3′ 5′-GCAAGAACTTTATTCAAAGTGCAA-3′

## Data Availability

Original data are available from the corresponding author upon request.

## References

[1] Mirzoev T.M., Shenkman B.S. (2018). Regulation of Protein Synthesis in Inactivated Skeletal Muscle: Signal Inputs, Protein Kinase Cascades, and Ribosome Biogenesis. Biochemistry.

[2] Ingalls C.P., Warren G.L., Armstrong R.B. (1999). Intracellular Ca^2+^ transients in mouse soleus muscle after hindlimb unloading and reloading. J. Appl. Physiol..

[3] Zaripova K.A., Kalashnikova E.P., Belova S.P., Kostrominova T.Y., Shenkman B.S., Nemirovskaya T.L. (2021). Role of Pannexin 1 ATP-Permeable Channels in the Regulation of Signaling Pathways during Skeletal Muscle Unloading. Int. J. Mol. Sci..

[4] Shenkman B.S., Nemirovskaya T.L. (2008). Calcium-dependent signaling mechanisms and soleus fiber remodeling under gravitational unloading. J. Muscle Res. Cell Motil..

[5] Kravtsova V.V., Matchkov V.V., Bouzinova E.V., Vasiliev A.N., Razgovorova I.A., Heiny J.A., Krivoi I.I. (2015). Isoform-specific Na,K-ATPase alterations precede disuse-induced atrophy of rat soleus muscle. BioMed Res. Int..

[6] Kravtsova V.V., Petrov A.M., Matchkov V.V., Bouzinova E.V., Vasiliev A.N., Benziane B., Zefirov A.L., Chibalin A.V., Heiny J.A., Krivoi I.I. (2016). Distinct alpha2 Na,K-ATPase membrane pools are differently involved in early skeletal muscle remodeling during disuse. J. Gen. Physiol..

[7] Casas M., Buvinic S., Jaimovich E. (2014). ATP signaling in skeletal muscle: From fiber plasticity to regulation of metabolism. Exerc. Sport. Sci. Rev..

[8] Nemirovskaya T.L., Sharlo K.A. (2022). Roles of ATP and SERCA in the Regulation of Calcium Turnover in Unloaded Skeletal Muscles: Current View and Future Directions. Int. J. Mol. Sci..

[9] Liu B., Cao W., Li J., Liu J. (2018). Lysosomal exocytosis of ATP is coupled to P2Y2 receptor in marginal cells in the stria vascular in neonatal rats. Cell Calcium.

[10] Vilchinskaya N.A., Mochalova E.P., Paramonova I., Belova S.P., Mirzoev T., Shenkman B. (2020). The Effect of β-GPA on the Markers of Anabolic and Catabolic Signaling Pathways in Rat Soleus Muscle at the Initial Stage of Hindlimb Unloading. Biochemistry.

[11] Diaz-Vegas A., Eisner V., Jaimovich E. (2019). Skeletal muscle excitation-metabolism coupling. Arch. Biochem. Biophys..

[12] Buvinic S., Almarza G., Bustamante M., Casas M., Lopez J., Riquelme M., Saez J.C., Huidobro-Toro J.P., Jaimovich E. (2009). ATP released by electrical stimuli elicits calcium transients and gene expression in skeletal muscle. J. Biol. Chem..

[13] Zaripova K.A., Belova S.P., Kostrominova T.Y., Shenkman B.S., Nemirovskaya T.L. (2024). P2Y1 and P2Y2 receptors differ in their role in the regulation of signaling pathways during unloading-induced rat soleus muscle atrophy. Arch. Biochem. Biophys..

[14] May C., Weigl L., Karel A., Hohenegger M. (2006). Extracellular ATP activates ERK1/ERK2 via a metabotropic P2Y1 receptor in a Ca^2+^ independent manner in differentiated human skeletal muscle cells. Biochem. Pharmacol..

[15] Bony C., Roche S., Shuichi U., Sasaki T., Crackower M.A., Penninger J., Mano H., Puceat M. (2001). A specific role of phosphatidylinositol 3-kinase gamma. A regulation of autonomic Ca^2+^ oscillations in cardiac cells. J. Cell Biol..

[16] Jaconi M., Bony C., Richards S.M., Terzic A., Arnaudeau S., Vassort G., Puceat M. (2000). Inositol 1,4,5-trisphosphate directs Ca^2+^ flow between mitochondria and the Endoplasmic/Sarcoplasmic reticulum: A role in regulating cardiac autonomic Ca^2+^ spiking. Mol. Biol. Cell.

[17] Rossi A.M., Taylor C.W. (2018). IP(3) receptors—lessons from analyses ex cellula. J. Cell Sci..

[18] Jaimovich E., Reyes R., Liberona J.L., Powell J.A. (2000). IP(3) receptors, IP(3) transients, and nucleus-associated Ca^2+^ signals in cultured skeletal muscle. Am. J. Physiol. Cell Physiol..

[19] Eltit J.M., Garcia A.A., Hidalgo J., Liberona J.L., Chiong M., Lavandero S., Maldonado E., Jaimovich E. (2006). Membrane electrical activity elicits inositol 1,4,5-trisphosphate-dependent slow Ca^2+^ signals through a Gbetagamma/phosphatidylinositol 3-kinase gamma pathway in skeletal myotubes. J. Biol. Chem..

[20] Cardenas C., Liberona J.L., Molgo J., Colasante C., Mignery G.A., Jaimovich E. (2005). Nuclear inositol 1,4,5-trisphosphate receptors regulate local Ca^2+^ transients and modulate cAMP response element binding protein phosphorylation. J. Cell Sci..

[21] Casas M., Altamirano F., Jaimovich E. (2012). Measurement of calcium release due to inositol trisphosphate receptors in skeletal muscle. Methods Mol. Biol..

[22] Arias-Calderon M., Almarza G., Diaz-Vegas A., Contreras-Ferrat A., Valladares D., Casas M., Toledo H., Jaimovich E., Buvinic S. (2016). Characterization of a multiprotein complex involved in excitation-transcription coupling of skeletal muscle. Skelet. Muscle.

[23] Powell J.A., Carrasco M.A., Adams D.S., Drouet B., Rios J., Muller M., Estrada M., Jaimovich E. (2001). IP(3) receptor function and localization in myotubes: An unexplored Ca^2+^ signaling pathway in skeletal muscle. J. Cell Sci..

[24] Araya R., Liberona J.L., Cardenas J.C., Riveros N., Estrada M., Powell J.A., Carrasco M.A., Jaimovich E. (2003). Dihydropyridine receptors as voltage sensors for a depolarization-evoked, IP3R-mediated, slow calcium signal in skeletal muscle cells. J. Gen. Physiol..

[25] Schiaffino S., Dyar K.A., Ciciliot S., Blaauw B., Sandri M. (2013). Mechanisms regulating skeletal muscle growth and atrophy. FEBS J..

[26] Vanhaesebroeck B., Ali K., Bilancio A., Geering B., Foukas L.C. (2005). Signalling by PI3K isoforms: Insights from gene-targeted mice. Trends Biochem. Sci..

[27] Vanhaesebroeck B., Vogt P.K., Rommel C. (2010). PI3K: From the bench to the clinic and back. Curr. Top. Microbiol. Immunol..

[28] Gulluni F., Martini M., De Santis M.C., Campa C.C., Ghigo A., Margaria J.P., Ciraolo E., Franco I., Ala U., Annaratone L. (2017). Mitotic Spindle Assembly and Genomic Stability in Breast Cancer Require PI3K-C2alpha Scaffolding Function. Cancer Cell.

[29] Devereaux K., Dall’Armi C., Alcazar-Roman A., Ogasawara Y., Zhou X., Wang F., Yamamoto A., De Camilli P., Di Paolo G. (2013). Regulation of mammalian autophagy by class II and III PI 3-kinases through PI3P synthesis. PLoS ONE.

[30] Hirsch E., Braccini L., Ciraolo E., Morello F., Perino A. (2009). Twice upon a time: PI3K’s secret double life exposed. Trends Biochem. Sci..

[31] Bilanges B., Posor Y., Vanhaesebroeck B. (2019). PI3K isoforms in cell signalling and vesicle trafficking. Nat. Rev. Mol. Cell Biol..

[32] Osorio-Fuentealba C., Contreras-Ferrat A.E., Altamirano F., Espinosa A., Li Q., Niu W., Lavandero S., Klip A., Jaimovich E. (2013). Electrical stimuli release ATP to increase GLUT4 translocation and glucose uptake via PI3Kgamma-Akt-AS160 in skeletal muscle cells. Diabetes.

[33] Yang H., Wang H., Pan F., Guo Y., Cao L., Yan W., Gao Y. (2023). New Findings: Hindlimb Unloading Causes Nucleocytoplasmic Ca^2+^ Overload and DNA Damage in Skeletal Muscle. Cells.

[34] Shenkman B.S. (2020). How Postural Muscle Senses Disuse? Early Signs and Signals. Int. J. Mol. Sci..

[35] Morello F., Perino A., Hirsch E. (2009). Phosphoinositide 3-kinase signalling in the vascular system. Cardiovasc. Res..

[36] Sharlo K.A., Lvova I.D., Tyganov S.A., Zaripova K.A., Belova S.P., Kostrominova T.Y., Shenkman B.S., Nemirovskaya T.L. (2023). The Effect of SERCA Activation on Functional Characteristics and Signaling of Rat Soleus Muscle upon 7 Days of Unloading. Biomolecules.

[37] Bodine S.C., Baehr L.M. (2014). Skeletal muscle atrophy and the E3 ubiquitin ligases MuRF1 and MAFbx/atrogin-1. Am. J. Physiol. Endocrinol. Metab..

[38] Baehr L.M., Hughes D.C., Waddell D.S., Bodine S.C. (2022). SnapShot: Skeletal muscle atrophy. Cell.

[39] Oudit G.Y., Kassiri Z. (2007). Role of PI3 kinase gamma in excitation-contraction coupling and heart disease. Cardiovasc. Hematol. Disord. Drug Targets.

[40] Kawano F., Ishihara A., Ohira Y. (2002). Air-righting responses to chronic hindlimb suspension and ambulation recovery in adult rats. Biol. Sci. Space.

[41] Ito N., Ruegg U.T., Takeda S. (2018). ATP-Induced Increase in Intracellular Calcium Levels and Subsequent Activation of mTOR as Regulators of Skeletal Muscle Hypertrophy. Int. J. Mol. Sci..

[42] Gomes D.A., Leite M.F., Bennett A.M., Nathanson M.H. (2006). Calcium signaling in the nucleus. Can. J. Physiol. Pharmacol..

[43] Park S., Scheffler T.L., Gerrard D.E. (2011). Chronic high cytosolic calcium decreases AICAR-induced AMPK activity via calcium/calmodulin activated protein kinase II signaling cascade. Cell Calcium.

[44] Witczak C.A., Sharoff C.G., Goodyear L.J. (2008). AMP-activated protein kinase in skeletal muscle: From structure and localization to its role as a master regulator of cellular metabolism. Cell. Mol. Life Sci..

[45] Raney M.A., Turcotte L.P. (2008). Evidence for the involvement of CaMKII and AMPK in Ca^2+^-dependent signaling pathways regulating FA uptake and oxidation in contracting rodent muscle. J. Appl. Physiol..

[46] Nakanishi A., Hatano N., Fujiwara Y., Sha’ri A., Takabatake S., Akano H., Kanayama N., Magari M., Nozaki N., Tokumitsu H. (2017). AMP-activated protein kinase-mediated feedback phosphorylation controls the Ca^2+^/calmodulin (CaM) dependence of Ca^2+^/CaM-dependent protein kinase kinase. J. Biol. Chem..

[47] Chin E.R. (2004). The role of calcium and calcium/calmodulin-dependent kinases in skeletal muscle plasticity and mitochondrial biogenesis. Proc. Nutr. Soc..

[48] Fajardo V.A., Rietze B.A., Chambers P.J., Bellissimo C., Bombardier E., Quadrilatero J., Tupling A.R. (2017). Effects of sarcolipin deletion on skeletal muscle adaptive responses to functional overload and unload. Am. J. Physiol. Cell Physiol..

[49] Sharlo K., Paramonova I., Turtikova O., Tyganov S., Shenkman B. (2019). Plantar mechanical stimulation prevents calcineurin-NFATc1 inactivation and slow-to-fast fiber type shift in rat soleus muscle under hindlimb unloading. J. Appl. Physiol..

[50] Mochalova E.P., Belova S.P., Kostrominova T.Y., Shenkman B.S., Nemirovskaya T.L. (2020). Differences in the Role of HDACs 4 and 5 in the Modulation of Processes Regulating MAFbx and MuRF1 Expression during Muscle Unloading. Int. J. Mol. Sci..

[51] Kubokawa M., Nakamura K., Komagiri Y. (2011). Interaction between Calcineurin and Ca/Calmodulin Kinase-II in Modulating Cellular Functions. Enzym. Res..

[52] Wang X.W., Hu S.J., Liu L. (2017). Phosphorylation and acetylation modifications of FOXO3a: Independently or synergistically?. Oncol. Lett..

[53] Belova S.P., Kalashnikova E.P., Tyganov S.A., Kostrominova T.Y., Shenkman B.S., Nemirovskaya T.L. (2022). Effect of enhanced muscle tone on the expression of atrogenes and cytoskeletal proteins during postural muscle unloading. Arch. Biochem. Biophys..

[54] You J.S., Dooley M.S., Kim C.R., Kim E.J., Xu W., Goodman C.A., Hornberger T.A. (2018). A DGK zeta-FoxO-ubiquitin proteolytic axis controls fiber size during skeletal muscle remodeling. Sci. Signal..

[55] Yudushkin I. (2019). Getting the Akt Together: Guiding Intracellular Akt Activity by PI3K. Biomolecules.

[56] Belova S.P., Vilchinskaya N.A., Mochalova E.P., Mirzoev T.M., Nemirovskaya T.L., Shenkman B.S. (2019). Elevated p70S6K phosphorylation in rat soleus muscle during the early stage of unloading: Causes and consequences. Arch. Biochem. Biophys..

[57] Rhee S.G. (2001). Regulation of phosphoinositide-specific phospholipase C. Annu. Rev. Biochem..

[58] Shenkman B.S., Belova S.P., Lomonosova Y.N., Kostrominova T.Y., Nemirovskaya T.L. (2015). Calpain-dependent regulation of the skeletal muscle atrophy following unloading. Arch. Biochem. Biophys..

[59] Glass D.J. (2003). Signalling pathways that mediate skeletal muscle hypertrophy and atrophy. Nat. Cell Biol..

[60] Baek M.O., Ahn C.B., Cho H.J., Choi J.Y., Son K.H., Yoon M.S. (2019). Simulated microgravity inhibits C2C12 myogenesis via phospholipase D2-induced Akt/FOXO1 regulation. Sci. Rep..

[61] Amar-Schwartz A., Ben Hur V., Jbara A., Cohen Y., Barnabas G.D., Arbib E., Siegfried Z., Mashahreh B., Hassouna F., Shilo A. (2022). S6K1 phosphorylates Cdk1 and MSH6 to regulate DNA repair. Elife.

[62] Han B., Zhu M.J., Ma C., Du M. (2007). Rat hindlimb unloading down-regulates insulin like growth factor-1 signaling and AMP-activated protein kinase, and leads to severe atrophy of the soleus muscle. Appl. Physiol. Nutr. Metab..

[63] Kaul G., Pattan G., Rafeequi T. (2011). Eukaryotic elongation factor-2 (eEF2): Its regulation and peptide chain elongation. Cell Biochem. Funct..

[64] Sharlo K., Tyganov S.A., Tomilovskaya E., Popov D.V., Saveko A.A., Shenkman B.S. (2021). Effects of Various Muscle Disuse States and Countermeasures on Muscle Molecular Signaling. Int. J. Mol. Sci..

[65] Baldwin K.M., Haddad F., Pandorf C.E., Roy R.R., Edgerton V.R. (2013). Alterations in muscle mass and contractile phenotype in response to unloading models: Role of transcriptional/pretranslational mechanisms. Front. Physiol..

[66] Kilkenny C., Browne W.J., Cuthill I.C., Emerson M., Altman D.G. (2010). Improving bioscience research reporting: The ARRIVE guidelines for reporting animal research. PLoS Biol..

[67] Grundy D. (2015). Principles and standards for reporting animal experiments in The Journal of Physiology and Experimental Physiology. Exp. Physiol..

[68] Morey-Holton E., Globus R.K., Kaplansky A., Durnova G. (2005). The hindlimb unloading rat model: Literature overview, technique update and comparison with space flight data. Adv. Space Biol. Med..

[69] Morey-Holton E.R., Globus R.K. (2002). Hindlimb unloading rodent model: Technical aspects. J. Appl. Physiol..

